# Increase in birthweight coverage of neonatal deaths is needed to monitor low birthweight prevalence in India: lessons from the National Family Health Survey

**DOI:** 10.1186/s12884-023-05865-2

**Published:** 2023-07-29

**Authors:** Rakhi Dandona, Arpita Paul, G. Anil Kumar

**Affiliations:** 1 Public Health Foundation of India, Gurugram, Haryana India; 2 Institute for Health Metrics and Evaluation, University of Washington, Seattle, USA

**Keywords:** Birthweight, Heaping, Low birthweight, India, NFHS, Nutrition, Target, Quality

## Abstract

**Background:**

Low birthweight (LBW), defined as birthweight < 2500gms, is the largest contributor to the malnutrition disability-adjusted-live-years in India. We report on the inadequacy of birthweight data, which is a significant barrier in the understanding of LBW epidemiology, to address malnutrition in India.

**Methods:**

Data from the recent round of the National Family Survey (NFHS-5) were utilised. Birthweight of livebirths in the last 5 years was documented in grams either from the health card or based on mother’s recall. We computed the coverage of birthweight measurement availability and the extent of heaping (values of 2500, 3000 and 3500gms) by the place of delivery and by the survival of newborn during the neonatal period. Heaping of > 55% was considered as poor-quality birthweight data. LBW prevalence per 100 livebirths was estimated and extrapolated for under-reporting of birthweight. Findings are reported for India and its 30 states.

**Results:**

Birthweight measurement coverage irrespective of the place of delivery was (89·8%; 95% CI 89·7–90) for India, and varied by 2 times among the states with the highest coverage in Tamil Nadu (99·3%) and the lowest in Nagaland (49·7%). Home deliveries had the least coverage of birthweight measurement (49.6%; 95% CI 49.0–50.1) as compared with public health facility (96.3%; 95% CI 96.2–96.3) and private health facility (96%; 95% CI 95.8–96.1) deliveries. This coverage was 66·5% (95% CI 65·2–67·7) among neonatal deaths as compared with 90.4 (95% CI 90.3–90.6) for livebirths who survived the neonatal period for India. The proportion of health card as the data source increased for livebirths born in year 2015 to year 2020 but then dropped for livebirths born in year 2021 (*p* < 0.001). The proportion of heaping was 52·0% (95% CI 51·7–52·2) in the recorded birthweight for India, and heaping > 55% was seen in 10 states irrespective of the type data source; and 3 states in addition had heaping > 55% in mother’s recall. LBW prevalence was estimated at 17·4% (95% CI 17·3–17·6) for India, and ranged from 4.5% in Nagaland and Mizoram to 22.5% in Punjab for livebirths for whom birthweight was available. We estimated LBW at 77.8% for whom birthweight was not available, and the adjusted LBW prevalence for all livebirths was estimated at 23.5% (95% CI 23.3–23.8) for India.

**Conclusions:**

Without measuring birthweight for every newborn irrespective of the survival and place of delivery, India may not able to address reduction in low birthweight and neonatal mortality effectively to meet global or national targets.

**Supplementary Information:**

The online version contains supplementary material available at 10.1186/s12884-023-05865-2.

## Background

Birthweight measurement is an important baseline from which growth for all newborns is measured [1]. With a prevalence of 21%, low birth weight (LBW) defined as birthweight < 2500gms, was the largest contributor to the malnutrition disability-adjusted-live-years in India, [2] and accounted for 83% of all neonatal deaths in 2017 [3]. A reduction of 30% in LBW prevalence between 2012 and 2030 is targeted to achieve as per the Global nutrition target [4], and a reduction of 6% was targeted by 2022 as per the India national nutrition target [5]. However, with only a modest declining trend in LBW prevalence seen from 1990–2017 in India, it is projected that India is unlikely to meet the LBW global nutrition target by 2025 [2, 6].

One of the most significant barriers in the understanding of LBW epidemiology is the inadequate quality of birthweight data in India, like in many other developing country settings [2, 7–9]. The population-level LBW prevalence trends for India are available from the National Family Health Survey (NFHS), which is an equivalent of the Demographic Health Survey (DHS) [10, 11]. Heaping in the birthweight data as reported in the NFHS has been of concern [7], and these data were not utilised in the global report on estimation of LBW prevalence [7, 8, 12]. Population-based survey data are often modelled with statistical methods to adjust for underreporting and misreporting of birth weight to estimate LBW prevalence [7, 13]. In this background, the aim of this report is to provide a nuanced understanding of what can be learnt based on the birthweight reporting in the most recent round of NFHS that could facilitate specific action to improve the robustness of LBW prevalence estimates for India and its states. To this effect, we undertook a detailed review of the availability and quality of birthweight data in NFHS-5 disaggregated by the state, place of delivery, birth outcome, and by source of birthweight documentation. We estimated the LBW prevalence and extrapolated it for under-reporting to highlight the implications of non-availability of birthweight on LBW prevalence. Specific recommendations are made to improve the robustness of birthweight documentation to facilitate monitoring of LBW prevalence to achieve the global and national nutrition targets.

## Methods

The NFHS is planned under the oversight of India’s Ministry of Health and Family Welfare and is coordinated by the International Institute for Population Sciences, Mumbai, India as the nodal agency with support from ORC Macro of USA and other agencies [10]. The primary objective of NFHS is to provide essential data on reproductive health and family planning, along with some other vital estimates. We utilised data from the recent round, NFHS-5 (2019–21), detailed sampling and survey methods for which are described elsewhere [10]. Ever-married women aged 15–49 years responded to questions on a large variety of reproductive and child health topics, including birthweight of their livebirths in the last 5 years. The birthweight was recorded by asking the woman—“was the baby weighted at birth” and “how much did the child weigh”; birthweight was documented in grams either from the health card or based on mother’s recall if health card was not available [10].

We compared the coverage of birthweight measurement availability by the place of delivery, and by the survival of newborn during the neonatal period. Place of delivery was categorised as public sector health facility, private sector health facility, and home. NGO health facility was considered under private sector health facility (0.54% of all deliveries). The quality of birthweight data was defined using the criteria utilised in the global report of LBW prevalence [14, 15], with the quality considered poor if > 55% of all birthweight values fell on three values—2500gms, 3000gms, or 3500gms (defined as heaping). [7] We removed birthweight values of > 9,000gms from this analysis (11 cases; 0.005%) [16]. We report on the prevalence of heaping by place of delivery, birth outcome, and compare it between the birthweight documented from a health card or mother’s recall. Furthermore, we estimated LBW prevalence per 100 livebirths using livebirths for whom birthweight was available as the denominator. We explored the association of neonatal mortality with birthweight. Based on the difference in proportion of neonatal deaths between livebirths for whom birthweight was available versus those for whom birthweight was not available, we also report proportionately adjusted LBW prevalence in those with birthweight available to estimate the LBW prevalence in those with birthweight not available.

This analysis was carried out for India and its 30 states. We categorised the states into two groups based on their socioeconomic development status – less and more developed states. [17] The less developed states included eight empowered action group states as identified by the government of India (Bihar, Madhya Pradesh, Jharkhand, Rajasthan, Uttar Pradesh, Uttarakhand, Chhattisgarh, Odisha and, Assam) and the other seven north-eastern states, and, the rest were categorised as more developed states [17]. The state of Jammu and Kashmir was divided into two union territories in 2019, but we report findings for the undivided state of Jammu and Kashmir. We report 95% confidence interval (CI) for estimates where relevant. All the analysis was done using STATA 13, R-4·2·0, and MS Excel.

## Results

A total of 724,115 (96·9% participation) ever-married women aged 15–49 years reported data on 232,920 livebirths in NFHS-5. Birthweight measurement was reported for 209,266 (89·8%) livebirths, it was reported to have been measured but value was not provided for 4,296 (1.9%) livebirths, and it was reported to not being measured for 19,358 (8.3%) livebirths.

### Coverage of birthweight measurement

With birthweight measurement reported for 209,266 livebirths, birthweight measurement coverage irrespective of the place of delivery was (89·8%; 95% CI 89·7–90), 96·1% (95% CI 95·9–96·2), and 86·4% (95% CI: 86·4–86·7) for India, the more and less developed states, respectively (Table 1). It varied by 2 times among the states with the highest coverage in Tamil Nadu (99·3%) and the lowest in Nagaland (49·7%) as shown in Table 1. This coverage of birthweight measurement for home deliveries was estimated at 49.6% (95% CI 49.0–50.1), and this coverage was nearly universal for public health facility (96.3%; 95% CI 96.2–96.3) and private health facility deliveries (96%; 95% CI 95.8–96.1) for India. Almost no state-variation was seen in this coverage for health facility deliveries but the home delivery coverage ranged from 22.1% in Nagaland to 100% in Kerala (Table 1).

**Table 1:** Coverage of birthweight measurement for livebirths for India and its states by place of delivery. CI denotes confidence interval

	**All deliveries irrespective of place of delivery**		**Public health facility deliveries**		**Private health facility deliveries**		**Home deliveries**	
	**Number of livebirths**	**Coverage of birthweight measurement N (%; 95% CI)**	**Number of livebirths**	**Coverage of birthweight measurement N (%; 95% CI)**	**Number of livebirths**	**Coverage of birthweight measurement N (%; 95% CI)**	**Number of livebirths**	**Coverage of birthweight measurement N (%; 95% CI)**
**India**	**2,32,920**	**2,09,266 (89.8; 89.7–90.0)**	**1,50,299**	**1,44,655 (96.3; 96.2–96.3)**	**51,012**	**48,945 (96.0; 95.8–96.1)**	**31,609**	**15,666 (49.6; 49.0–50.1)**
**Less developed states**	**1,52,818**	**1,32,299 (86.4; 86.4–86.7)**	**1,01,522**	**97,178 (95.7; 95.6–95.9)**	**23,820**	**22,280 (93.5; 93.2–93.9)**	**27,476**	**12,841 (46.7; 46.2–47.3)**
Arunachal Pradesh	5,524	4,510 (81.6; 80.6–82.7)	4,177	4,002 (95.8; 95.2–96.4)	241	232 (96.3; 93.9–98.7)	1,106	276 (25.0; 22.4–27.5)
Assam	10,645	9,867 (92.7; 92.2–93.2)	7,991	7,803 (97.7; 97.3–98.0)	1,000	986 (98.6; 97.9–99.3)	1,654	1,078 (65.2; 62.9–67.5)
Bihar	21,040	16,236 (77.2; 76.6–77.7)	12,352	11,438 (92.6; 92.1–93.1)	3,935	3,510 (89.2; 88.2–90.2)	4,753	1,288 (27.1; 25.8–28.4)
Chhattisgarh	8,514	8,090 (95.0; 94.6–95.5)	6,097	5,982 (98.1; 97.8–98.5)	1,068	1,043 (97.7; 96.8–98.6)	1,349	1,065 (79.0; 76.8–81.1)
Jharkhand	10,047	8,599 (85.6; 84.9–86.3)	5,938	5,712 (96.2; 95.7–96.7)	1,667	1,621 (97.2; 96.5–98.0)	2,442	1,266 (51.8; 49.9–53.8)
Madhya Pradesh	16,280	15,158 (93.1; 92.7–93.5)	13,212	12,796 (96.9; 96.6–97.1)	1,474	1,436 (97.4; 96.6–98.2)	1,594	926 (58.1; 55.7–60.5)
Manipur	3,225	2,420 (75.0; 73.5–76.5)	1,619	1,571 (97.0; 96.2–97.9)	577	561 (97.2; 95.9–98.6)	1,029	288 (28.0; 25.2–30.7)
Meghalaya	6,628	5,476 (82.6; 81.7–83.5)	3,153	3,044 (96.5; 95.9–97.2)	534	513 (96.1; 94.4–97.7)	2,941	1,919 (65.3; 63.5–67.0)
Mizoram	2,454	2,212 (90.1; 89.0–91.3)	1,781	1,743 (97.9; 97.2–98.5)	188	188 (100.0)	485	281 (57.9; 53.5–62.3)
Nagaland	3,052	1,518 (49.7; 48.0–51.5)	1,042	943 (90.5; 88.7–92.3)	186	172 (92.5; 88.7–96.3)	1,824	403 (22.1; 20.2–24.0)
Odisha	8,522	8,332 (97.8; 97.5–98.1)	6,779	6,697 (98.8; 98.5–99.1)	1,022	1,013 (99.1; 98.5–99.7)	721	622 (86.3; 83.8–88.8)
Rajasthan	14,643	13,779 (94.1; 93.7–94.5)	11,566	11,150 (96.4; 96.1–96.7)	2,356	2,287 (97.1; 96.4–97.8)	721	342 (47.4; 43.8–51.1)
Sikkim	620	607 (97.9; 96.8–99.0)	525	515 (98.1; 96.9–99.3)	71	70 (98.6; 95.8–101.3)	24	22 (91.7; 80.4–102.9)
Tripura	2,074	1,847 (89.1; 87.7–90.4)	1,597	1,534 (96.1; 95.1–97.0)	221	212 (95.9; 93.3–98.5)	256	101 (39.5; 33.5–45.5)
Uttar Pradesh	35,766	30,370 (84.9; 84.5–85.3)	21,355	20,020 (93.8; 93.4–94.1)	8,494	7,683 (90.5; 89.8–91.1)	5,917	2,667 (45.1; 43.8–46.3)
Uttarakhand	3,784	3,278 (86.6; 85.5–87.7)	2,338	2,228 (95.3; 94.4–96.2)	786	753 (95.8; 94.4–97.2)	660	297 (45.0; 41.2–48.8)
**More developed states**	**77,101**	**74,067 (96.1; 95.9–96.2)**	**46,525**	**45,287 (97.3; 97.2–97.5)**	**26,512**	**25,998 (98.1; 97.9–98.2)**	**4,064**	**2,782 (68.5; 67.0–69.9)**
Andhra Pradesh	2,833	2,780 (98.1; 97.6–98.6)	1,421	1,405 (98.9; 98.3–99.4)	1,320	1,301 (98.6; 97.9–99.2)	92	74 (80.4; 72.3–88.6)
Delhi	2,937	2,756 (93.8; 93.0–94.7)	1,827	1,765 (96.6; 95.8–97.4)	865	837 (96.8; 95.6–97.9)	245	154 (62.9; 56.8–68.9)
Goa	369	366 (99.2; 98.3–100.1)	214	212 (99.1; 97.8–100.3)	154	154 (100.0)	1	0
Gujarat	9,868	9,529 (96.6; 96.2–96.9)	4,457	4,353 (97.7; 97.2–98.1)	4,710	4,622 (98.1; 97.7–98.5)	701	554 (79.0; 76.0–82.0)
Haryana	6,915	6,505 (94.1; 93.5–94.6)	4,027	3,891 (96.6; 96.1–97.2)	2,502	2,412 (96.4; 95.7–97.1)	386	202 (52.3; 47.3–57.3)
Himachal Pradesh	2,635	2,498 (94.8; 94.0–95.6)	1,875	1,833 (97.8; 97.1–98.4)	434	430 (99.1; 98.2–100.0)	326	235 (72.1; 67.2–77.0)
Jammu and Kashmir	5,857	5,243 (89.5; 88.7–90.3)	5,038	4,710 (93.5; 92.8–94.2)	288	275 (95.5; 93.1–97.9)	531	258 (48.6; 44.3–52.8)
Karnataka	8,383	8,182 (97.6; 97.3–97.9)	5,642	5,548 (98.3; 98.0–98.7)	2,487	2,438 (98.0; 97.5–98.6)	254	196 (77.2; 72.0–82.3)
Kerala	2,734	2,710 (99.1; 98.8–99.5)	961	950 (98.9; 98.2–99.5)	1,768	1,755 (99.3; 98.9–99.7)	5	5 (100)
Maharashtra	9,520	9,140 (96.0; 95.6–96.4)	5,830	5,676 (97.4; 96.9–97.8)	3,144	3,091 (98.3; 97.9–98.8)	546	373 (68.3; 64.4–72.2)
Punjab	5,616	5,319 (94.7; 94.1–95.3)	3,129	3,006 (96.1; 95.4–96.8)	2,211	2,135 (96.6; 95.8–97.3)	276	178 (64.5; 58.8–70.1)
Tamil Nadu	6,498	6,454 (99.3; 99.1–99.5)	4,357	4,329 (99.4; 99.1–99.6)	2,115	2,103 (99.4; 99.1–99.8)	26	22 (84.6; 70.5–98.8)
Telangana	7,318	7,177 (98.1; 97.8–98.4)	3,647	3,596 (98.6; 98.2–99.0)	3,464	3,412 (98.5; 98.1–98.9)	207	169 (81.6; 76.4–86.9)
West Bengal	5,618	5,408 (96.3; 95.8–96.8)	4,100	4,013 (97.9; 97.4–98.3)	1,050	1,033 (98.4; 97.6–99.1)	468	362 (77.4; 73.6–81.1)

**Fig. 1:**
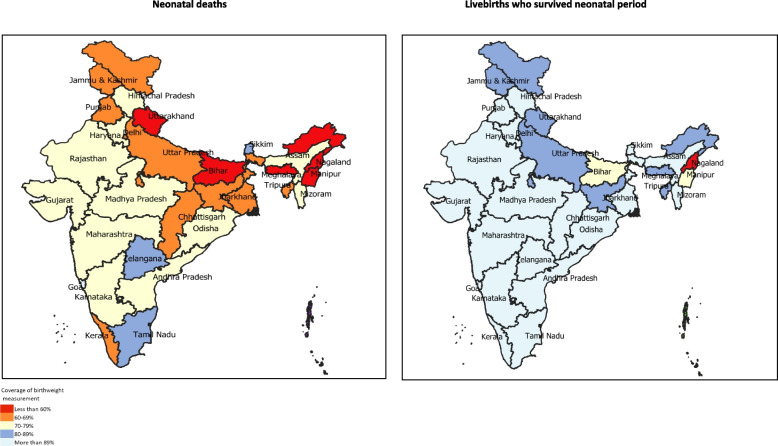
State-level coverage of birthweight measurement for livebirths by survival during the neonatal period

The coverage of birthweight measurement by survival during the neonatal period was significantly different (Fig. 1 and Additional file 1). This coverage was 66·5% (95% CI 65·2–67·7) among neonatal deaths, which was 1·4 times less as compared with the livebirths who survived the neonatal period (90.4; 95% CI 90.3–90.6) for India. The coverage of birthweight measurement among neonatal deaths varied by 3 times among the states with the highest coverage in Goa (100%) and the lowest in Nagaland (33·3%) as shown in Fig. 1.

**Fig. 2:**
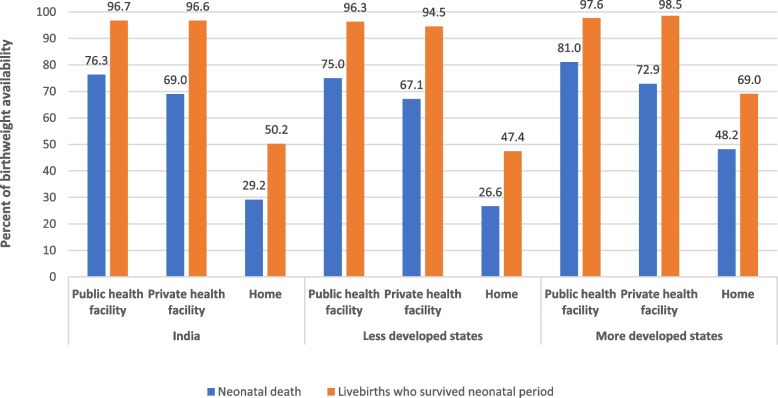
Distribution of availability of birthweight by survival outcome during the neonatal period for India and for states categorised by development status

The coverage of birthweight measurement among neonatal deaths was significantly lower as compared with the livebirths who survived the neonatal period irrespective of the place of delivery, though the gap in this coverage was the highest for home deliveries (Fig. 2).

### Source of birthweight data

Among the 209,266 livebirths who had birthweight available, the source of birthweight was health card for 124,365 (59.4%) and was mother’s recall for the remaining 84,901 (40.6%) livebirths. Health card as the source ranged from 40.2% in Delhi to 74% in Odisha for livebirths delivered in public health facilities, 36.8% in Bihar to 81.2% in West Bengal for livebirths delivered in private health facilities, and 32.4% in Andhra Pradesh to 77.3% in Assam, Tamil Nadu and West Bengal for those delivered at home (Additional file 2). The proportion of health card as the data source increased for livebirths born in year 2015 to year 2020 but then dropped for livebirths born in year 2021 (*p* < 0.001; Additional file 3).

### Quality of birthweight data

**Fig. 3:**
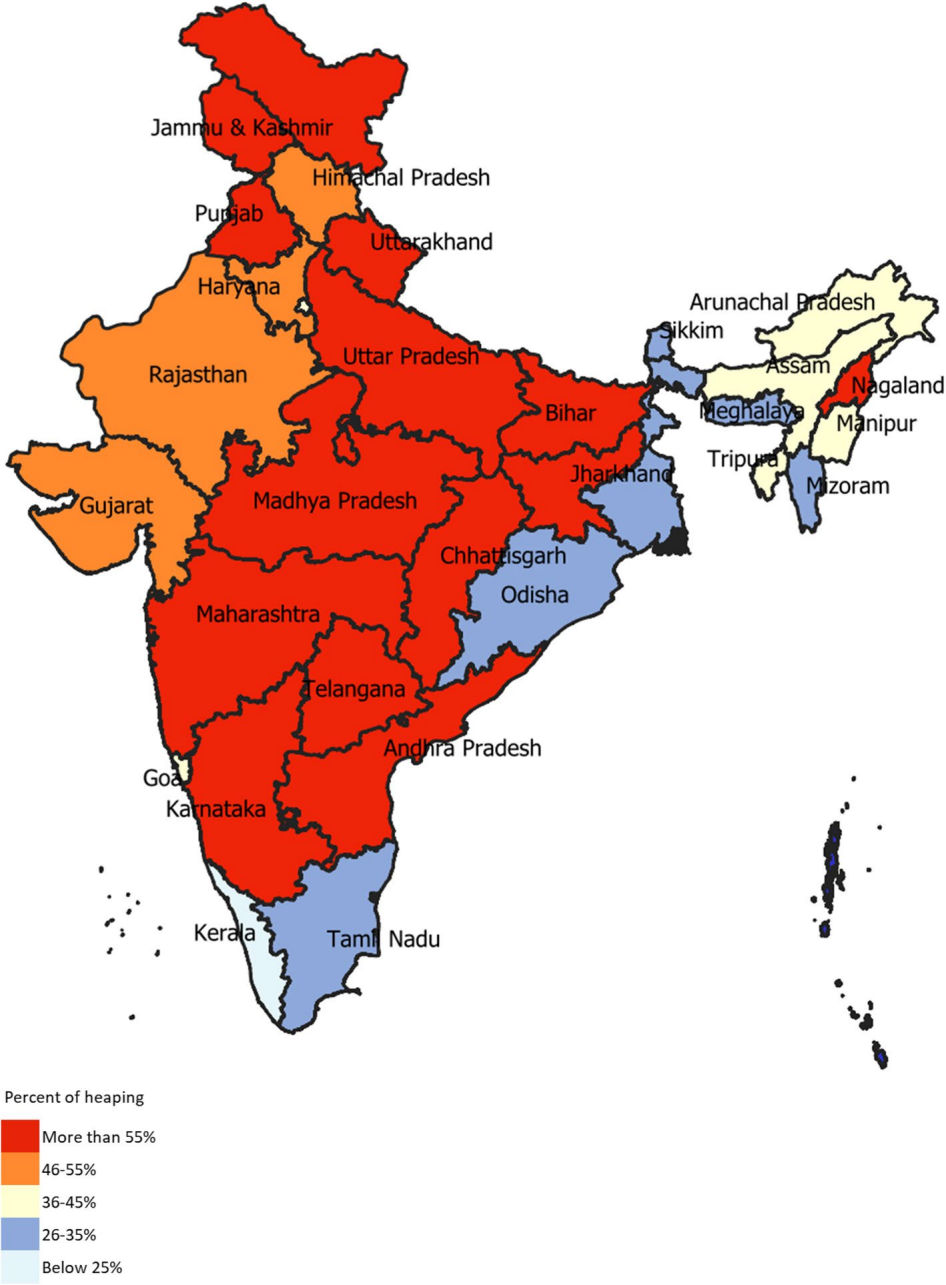
Percent of heaping in the recorded birthweight by state

The proportion of heaping was 52·0% (95% CI 51·7–52·2) in the recorded birthweight for India, and 52.4% in the less developed and 51.5% in the more developed states (Additional file 4). Recording of birthweight at 3,000gms was favored over 2,500gms in most states. A total of 13 (43.3%) of the 30 states had heaping > 55% indicating poor quality of birthweight data (Fig. 3 and Additional file 5).

Considering the source of birthweight data, heaping > 55% was seen in 10 states irrespective of the type data source; and 3 states in addition had heaping > 55% in mother’s recall. There was a significant correlation with the proportion of heaping reducing with increase in the proportion of health record as the source of data (*r* =—0.381, *p* = 0.038) whereas a reverse correlation was seen with the mother’s recall as the source (*r* = 0.421, *p* = 0.021; Additional file 6). By the place of delivery, 2500gms as the birthweight was reported by 19.9%, 18.4% and 20.9% of livebirths delivered in public health facility, private health facility, and home delivery (*p* < 0.001). Recording of 3000gms was reported for 24%, 22.3% and 25.6% of livebirths delivered in public health facility, private health facility, and home delivery (*p* < 0.001).

### Prevalence of LBW

The LBW prevalence per 100 livebirths was estimated at 17·4% (95% CI 17·3–17·6) for India, and ranged from 4.5% in Nagaland and Mizoram to 22.5% in Punjab (Table 2). On considering livebirths by survival during the neonatal period, the LBW prevalence among neonatal deaths was estimated 2.34 times higher (39.8%; 95% CI 38.3–41.4) as compared with LBW prevalence among those who survived the neonatal period (17.0%; 95% CI 16.8–17.2). The LBW prevalence among neonatal deaths ranged from 7.7% in Nagaland to 57.1% in Kerala.

**Table 2:** Prevalence of low birthweight (LBW) with and without adjustment, India and its states. CI denotes confidence interval

	**Livebirths with birthweight available**		**Livebirths with birthweight not available**		**Ratio of percent of neonatal deaths between birthweight not available and available**	**LBW prevalence per 100 livebirths** **(95% CI)**	**Adjusted LBW prevalence per 100 livebirths** **(95% CI)**	**Ratio of adjusted to unadjusted LBW prevalence per 100 livebirths**
	**Total**	**Number of neonatal deaths (% of total)**	**Total**	**Number of neonatal deaths (% of total)**				
**India**	**2,09,266**	**3,763 (1.8)**	**23,654**	**1,900 (8.0)**	**4.5**	**17.4 (17.2–17.6)**	**23.5 (23.3–23.8)**	**1.35**
**Less developed states**	**1,32,299**	**2,731 (2.1)**	**20,519**	**1,561 (7.6)**	**3.7**	**17.3 (17.1–17.6)**	**23.6 (23.3–23.9)**	**1.36**
Arunachal Pradesh	4,510	18 (0.4)	1,014	25 (2.5)	6.2	10.7 (9.6–11.8)	20.8 (19.5–22.1)	1.95
Assam	9,867	180 (1.8)	778	62 (8.0)	4.4	15.6 (14.7–16.4)	19.4 (18.5–20.3)	1.25
Bihar	16,236	399 (2.5)	4,804	337 (7.0)	2.9	16.9 (16.2–17.6)	24.0 (23.3–24.7)	1.42
Chhattisgarh	8,090	175 (2.2)	424	77 (18.2)	8.4	17.7 (16.6–18.7)	24.2 (23.0–25.3)	1.37
Jharkhand	8,599	170 (2.0)	1,448	111 (7.7)	3.9	15.7 (14.7–16.6)	22.2 (21.2–23.2)	1.41
Madhya Pradesh	15,158	358 (2.4)	1,122	131 (11.7)	4.9	20.2 (19.4–21.0)	25.7 (24.9–26.5)	1.27
Manipur	2,420	26 (1.1)	805	25 (3.1)	2.9	6.2 (5.1–7.4)	9.2 (8.0–10.4)	1.47
Meghalaya	5,476	46 (0.8)	1,152	78 (6.8)	8.1	11.8 (10.8–12.9)	26.4 (25.1–27.7)	2.23
Mizoram	2,212	17 (0.8)	242	5 (2.1)	2.7	4.5 (3.5–5.6)	5.3 (4.2–6.4)	1.17
Nagaland	1,518	13 (0.9)	1,534	26 (1.7)	2.0	4.5 (3.2–5.8)	6.7 (5.6–7.8)	1.49
Odisha	8,332	198 (2.4)	190	51 (26.8)	11.3	19.1 (18.1–20.2)	23.5 (22.4–24.6)	1.23
Rajasthan	13,779	247 (1.8)	864	67 (7.8)	4.3	18.6 (17.8–19.4)	22.2 (21.4–23.0)	1.20
Sikkim	607	4 (0.7)	13	1 (7.7)	11.7	8.1 (5.4–10.7)	9.9 (7.0–12.8)	1.22
Tripura	1,847	30 (1.6)	227	18 (7.9)	4.9	19.7 (17.5–21.9)	28.1 (25.7–30.4)	1.42
Uttar Pradesh	30,370	795 (2.6)	5,396	498 (9.2)	3.5	20.5 (20.0–21.1)	28.3 (27.8–28.9)	1.38
Uttarakhand	3,278	55 (1.7)	506	49 (9.7)	5.8	17.8 (16.2–19.4)	29.1 (27.3–30.9)	1.64
**More developed states**	**74,067**	**1,011 (1.4)**	**3,034**	**325 (10.7)**	**7.8**	**17.6 (17.3–18.0)**	**22.4 (22.0–22.7)**	**1.27**
Andhra Pradesh	2,780	41 (1.5)	53	14 (26.4)	17.9	16.7 (15.0–18.4)	22.0 (20.1–23.9)	1.32
Delhi	2,756	34 (1.2)	181	17 (9.4)	7.6	21.8 (19.9–23.7)	30.7 (28.7–32.7)	1.41
Goa	366	2 (0.5)	3	-	-	13.9 (9.6–18.3)	13.8 (9.5–18.1)	0.99
Gujarat	9,529	160 (1.7)	339	42 (12.4)	7.4	18.4 (17.5–19.4)	22.5 (21.5–23.5)	1.22
Haryana	6,505	108 (1.7)	410	44 (10.7)	6.5	20.5 (19.3–21.7)	27.1 (25.8–28.4)	1.32
Himachal Pradesh	2,498	41 (1.6)	137	11 (8.0)	4.9	14.5 (12.8–16.2)	17.4 (15.7–19.2)	1.20
Jammu and Kashmir	5,243	38 (0.7)	614	17 (2.8)	3.8	10.9 (9.9–12.0)	14.2 (13.1–15.3)	1.30
Karnataka	8,182	94 (1.1)	201	40 (19.9)	17.3	16.1 (15.1–17.1)	22.4 (21.3–23.5)	1.39
Kerala	2,710	7 (0.3)	24	4 (16.7)	64.5	16.3 (14.6–18.0)	25.4 (23.4–27.4)	1.56
Maharashtra	9,140	143 (1.6)	380	36 (9.5)	6.1	20.3 (19.3–21.3)	24.4 (23.3–25.5)	1.20
Punjab	5,319	87 (1.6)	297	38 (12.8)	7.8	22.3 (20.9–23.7)	30.4 (28.9–31.8)	1.36
Tamil Nadu	6,454	73 (1.1)	44	11 (25.0)	22.1	17.3 (16.2–18.4)	19.8 (18.6–21.0)	1.14
Telangana	7,177	121 (1.7)	141	22 (15.6)	9.3	13.7 (12.8–14.7)	15.9 (14.9–17.0)	1.16
West Bengal	5,408	62 (1.1)	210	29 (13.8)	12.0	18.8 (17.5–20.1)	26.5 (25.1–28.0)	1.41

The proportion of neonatal deaths in livebirths with birthweight not available (8%) was significantly higher than among those livebirths for whom birthweight was available (1.8%; *p* < 0.0001) for India (Table 2). Using the ratio of 4.5 higher neonatal deaths for India and assuming a direct correspondence between neonatal mortality rate and LBW, we estimated that LBW among livebirths for whom birthweight was not available would be 77.8%, that is 4.5 times higher than the 17.4% LBW among livebirths for whom birthweight was available. Based on the proportions of these two groups among all livebirths, we estimated an overall adjusted LBW of 23.5% (95% CI 23.3–23.8) among all livebirths for India. The ratio of neonatal deaths between those with birthweight not available and available ranged from 0 to 64.5 at the state-level (Table 2). With the adjustment, the ratio of adjusted to non-adjusted LBW prevalence ranged from 0.99 in Goa to 2.23 in Meghalaya.

## Discussion

Extrapolating the study findings, an estimated 18.7 million livebirths born between 2015–21 in India have no birthweight data with underreporting from newborns at the greatest risk for LBW leading to a potential underestimation of LBW prevalence. To track and achieve the global and national nutrition target of LBW reduction, India needs to invest in improving the coverage of birthweight measurement among livebirths who do not survive the neonatal period, and in the quality of birthweight measurement across most states irrespective of the place of delivery. LBW prevalence for India estimated was 17.4% considering only livebirths with birthweight available, and 23.5% in all livebirths by proportionately adjusting for those who did not birthweight available based on higher proportion of neonatal mortality in them. Despite the adjustment made for neonatal mortality being simplistic, the extent of variation in LBW prevalence with this adjustment conveys the enormous implications of non-availability of birthweight for the planning of appropriate interventions to reduce LBW in India.

One of the proposed newborn quality of care indicator at health-facility level in low- and middle-income setting is facility neonatal mortality rate disaggregated by birth weight [18]. In this sample, 92.5% of the livebirths were delivered in a health facility and majority of these were in public health facilities; however, birthweight was not available for 1 in 4 neonatal deaths in public health facilities and for 1 in 3 neonatal deaths in private health facilities. As the measurement of accurate birthweight for a newborn is important to enable provision of life-saving interventions [13], and in the context of LBW and short gestation being the predominant risk factors for neonatal mortality in India [3], ensuring birthweight is measured for all livebirths irrespective of survival at birth is extremely important. Urgent and sustained effort is needed to track neonatal mortality rate disaggregated by birthweight on a routine basis, which is currently not tracked in the Indian health management information system (HMIS) [12]. Furthermore, with 70% of all livebirths delivered in public health facility in this sample, the HMIS should be able to provide good quality birthweight data on a regular basis in addition to the population-based surveys such as the NFHS [10]. The birthweight documentation is rounded off instead of exact documentation, as evident by the proportion of extreme heaping, despite the availability of weighing scale being nearly universal in public health facilities [19] could limit the usefulness of HMIS to monitor LBW over time [12]. Despite true birthweights being normally distributed, heaping of birthweight measurement is common in developing country setting and birthweight rounding due to the “digit bias” for numbers ending in 0 or 5 is also known [20–23]. However, heaping at 2500gms has implications for LBW prevalence as it may result in LBW infants being misclassified as normal birthweight. Interestingly, we found a preference for 3000gms over 2500gms, which is different than that reported for the previous rounds of NFHS [12]. This change in preference is to be noted and its implications are to be explored further. Irrespective of the documentation as 2500 or 3000gms, this documentation reflects imprecision in the measurement which, in turn, could be a reflection of sub-optimal practices when measuring birthweight [24].

We found the quality of birthweight data to be poor in 13 of the 30 states, and surprisingly this poor quality was irrespective of the type data source in 10 of these 13 states. Health card was the data source for nearly 3 in 5 birthweights documented in NFHS for livebirths over the last 5 years but this changed to 1 in 2 for in the most recent year of 2021. The NFHS questionnaire instructions are to record the birthweight from the health card if available [25]. The increase in mother’s recall as the source in the most recent year could either indicate non-availability of the health card or adaptation in the process of documentation as response to Covid-19 pandemic.

It is important to note that birthweight is to be ideally measured within the first hours after birth before significant postnatal weight loss has occurred as term neonates lose between 3.5% and 6.6% of their birthweight within the first 2.5–2.7 days of life [26]. Therefore, if the birthweight measurement is delayed by a day or more, a newborn weighing over 2500 g may then weigh < 2500 g due to physiological weight loss. The population surveys, including the NFHS, capture birthweight using a generic question of “was the baby weighed at birth” without specifics of the exact timing when the birthweight was measured post birth [25]. The LBW working group has recommended to restrict ‘birthweight’ to a weight measured in the first 48 h of life, in the absence of which a weight measured during the first week of life could be classified as an ‘early neonatal weight’ but not ‘birthweight’ [27].

The India Newborn Action Plan aims to reduce LBW through improved preconception and antenatal care, adolescent-specific health services, nutritional counselling, and micronutrient supplementation [28], and India’s National Nutrition Mission had established annual target for reducing LBW by 6% in India by 2022 [5]. Improving the quality and coverage of birthweight reporting, including by strengthening national data monitoring and surveillance systems, will be critical to reduce LBW going forward. Specific guidance to precisely measure birthweight of all livebirths within the ideal time period is necessary. Furthermore, to improve both the birthweight coverage and accuracy in India, urgent efforts are needed to understand why the health providers do not document birthweight for all newborns irrespective of the survival, and why accurate birthweight measurement is done by them [29]. Such an understanding is needed for health providers across the public health facilities and private health facilities, and among those who assist with home births if the LBW tracking in India needs to be robust to improve birthweight availability and facilitate monitoring of malnutrition targets. In addition to birthweight data reported through routine administrative systems to be accurate and complete, such improvements in documentation will also likely strengthen the collection of birthweight data in household surveys.

Documentation of birthweight for some livebirths based on mother’s recall in the survey could be considered a limitation. Though the NFHS documents birthweight only for livebirths, we have previously documented birthweight non-availability at 85% for stillbirths in a state in India [30]. The strengths of our study include an attempt to estimate LBW for all livebirths at the population level by extrapolating for the under-reporting, and nuanced details by birth outcome, place of delivery and data source disaggregated by states that can facilitate actionable interventions or further implementation research to improve tracking of LBW, which is a priority global health indicator.

## Conclusion

Without accurately measuring birthweight for every newborn irrespective of the survival and place of delivery, India may not able to address reduction in low birthweight and neonatal mortality effectively to meet global or national targets.

## Supplementary Information

**Additional file 1.** Coverage of birthweight measurement for livebirths by survival during the neonatal period, India and its states, NFHS 5. CI denotes confidence interval.

**Additional file 2.** Prevalence of birthweight (BW) measurement recorded from health card for livebirths by place of delivery for India and its states, NFHS 5 by place of delivery. CI denotes confidence interval.

**Additional file 3.** Distribution of the data source for birthweight by the year of birth for livebirths, India.

**Additional file 4.** Percent of heaping (birthweight documented at 2500 or 3000 or 3500gms) in birthweight, India and its states, NFHS 5. CI denotes confidence interval.

**Additional file 5.** Percent of heaping (birthweight documented at 2500 or 3000 or 3500gms) by documentation source, India and its states, NFHS-5. CI denotes confidence interval.

**Additional file 6.** Correlation of heaping in birthweight (BW) by coverage of birthweight measurement from data source for India, NFHS 5 (2019-21).

## Data Availability

The data used in these analyses are available from the International Institute for Population Sciences on request (http://rchiips.org/NFHS/data1.shtml). The datasets analysed are available from the corresponding author on reasonable request.

## Abbreviations

CI: Confidence interval
HMIS: Health management information system
LBW: Low birth weight
NFHS: National Family Health Survey
