# Supplementary material for: Increase in birthweight coverage of neonatal deaths is needed to monitor low birthweight prevalence in India: lessons from the National Family Health Survey
Source: BMC Pregnancy Childbirth. 2023 Jul 29;23:545. doi: 10.1186/s12884-023-05865-2 (PMC10386228; doi:10.1186/s12884-023-05865-2)
Supplement: Supplementary file 1 — Additional file 1. Coverage of birthweight measurement for livebirths by survival during the neonatal period, India and its states, NFHS 5. CI denotes confidence interval. [file 12884_2023_5865_MOESM1_ESM.docx]

1. **Coverage of birthweight measurement for livebirths by survival during the neonatal period, India and its states, NFHS 5. CI denotes confidence interval.**

|  | **Neonatal deaths** | | **Livebirths who survived neonatal period** | |
| --- | --- | --- | --- | --- |
|  | **Number** | **Coverage of birthweight measurement**  **N (%; 95% CI)** | **Number** | **Coverage of birthweight measurement**  **N (%; 95% CI)** |
| **India** | **5,663** | **3,763 (66.5; 65.2-67.7)** | **227,257** | **205,503 (90.4; 90.3-90.6)** |
| **Less developed states** | **4,292** | **2,731 (63.3; 62.2-65.1)** | **148,526** | **129,568 (87.2; 87.1-87.4)** |
| Arunachal Pradesh | 43 | 18 (41.9; 26.9-56.8) | 5,481 | 4,492 (82.0; 80.9-83.0) |
| Assam | 242 | 180 (74.4; 68.9-79.9) | 10,403 | 9,687 (93.1; 92.6-93.6) |
| Bihar | 736 | 399 (54.2; 50.6-57.8) | 20,304 | 1,5837 (78.0; 77.4-78.6) |
| Chhattisgarh | 252 | 175 (69.4; 63.8-75.1) | 8,262 | 7,915 (95.8; 95.4-96.2) |
| Jharkhand | 281 | 170 (60.5; 54.8-66.2) | 9,766 | 8,429 (86.3; 85.6-87.0) |
| Madhya Pradesh | 489 | 358 (73.2; 69.3-77.1) | 15,791 | 14,800 (93.7; 93.4-94.1) |
| Manipur | 51 | 26 (51.0; 37.1-64.8) | 3,174 | 2,394 (75.4; 73.9-76.9) |
| Meghalaya | 124 | 46 (37.1; 28.6-45.6) | 6,504 | 5,430 (83.5; 82.6-84.4) |
| Mizoram | 22 | 17 (77.3; 59.4-95.2) | 2,432 | 2,195 (90.3; 89.1-91.4) |
| Nagaland | 39 | 13 (33.3; 18.3-48.3) | 3,013 | 1,505 (50.0; 48.2-51.7) |
| Odisha | 249 | 198 (79.5; 74.5-84.5) | 8,273 | 8,134 (98.3; 98.0-98.6) |
| Rajasthan | 314 | 247 (78.7; 74.1-83.2) | 14,329 | 13,532 (94.4; 94.1-94.8) |
| Sikkim | 5 | 4 (80.0; 40.8-119.0) | 615 | 603 (98.1; 97.0-99.1) |
| Tripura | 48 | 30 (62.5; 48.7-76.3) | 2,026 | 1,817 (89.7; 88.4-91.0) |
| Uttar Pradesh | 1,293 | 795 (61.5; 58.8-64.1) | 34,473 | 29,575 (85.8; 85.4-86.2) |
| Uttarakhand | 104 | 55 (52.9; 43.2-62.5) | 3,680 | 3,223 (87.6; 86.5-88.7) |
| **More developed states** | **1,336** | **1,011 (75.7; 73.4-78.0)** | **75,765** | **73,056 (96.4; 96.3-96.6)** |
| Andhra Pradesh | 55 | 41 (74.6; 62.9-86.2) | 2,778 | 2,739 (98.6; 98.2-99.0) |
| Delhi | 51 | 34 (66.7; 53.6-79.7) | 2,886 | 2,722 (94.3; 93.5-95.2) |
| Goa | 2 | 2 (100.0; 100.0-100.0) | 367 | 364 (99.2; 98.3-100.0) |
| Gujarat | 202 | 160 (79.2; 73.6-84.8) | 9,666 | 9,369 (96.9; 96.6-97.3) |
| Haryana | 152 | 108 (71.1; 63.8-78.3) | 6,763 | 6,397 (94.6; 94.1-95.1) |
| Himachal Pradesh | 52 | 41 (78.9; 67.6-90.1) | 2,583 | 2,457 (95.1; 94.3-96.0) |
| Jammu and Kashmir | 55 | 38 (69.1; 56.8-81.4) | 5,802 | 5,205 (89.7; 88.9-90.5) |
| Karnataka | 134 | 94 (70.2; 62.4-77.9) | 8,249 | 8,088 (98.1; 97.8-98.4) |
| Kerala | 11 | 7 (63.6; 33.8-93.5) | 2,723 | 2,703 (99.3; 99.0-100.0) |
| Maharashtra | 179 | 143 (79.9; 74.0-85.8) | 9,341 | 8,997 (96.3; 95.9-96.7) |
| Punjab | 125 | 87 (69.6; 61.5-77.7) | 5,491 | 5,232 (95.3; 94.7-95.8) |
| Tamil Nadu | 84 | 73 (86.9; 79.6-94.1) | 6,414 | 6,381 (99.5; 99.3-99.7) |
| Telangana | 143 | 121 (84.6; 78.7-90.6) | 7,175 | 7,056 (98.3; 98.1-98.6) |
| West Bengal | 91 | 62 (68.1; 58.5-77.8) | 5,527 | 5,346 (96.7; 96.3-97.2) |
