# Supplementary material for: Increase in birthweight coverage of neonatal deaths is needed to monitor low birthweight prevalence in India: lessons from the National Family Health Survey
Source: BMC Pregnancy Childbirth. 2023 Jul 29;23:545. doi: 10.1186/s12884-023-05865-2 (PMC10386228; doi:10.1186/s12884-023-05865-2)
Supplement: Supplementary file 2 — Additional file 2. Prevalence of birthweight (BW) measurement recorded from health card for livebirths by place of delivery for India and its states, NFHS 5 by place of delivery. CI denotes confidence interval. [file 12884_2023_5865_MOESM2_ESM.docx]

1. **Prevalence of birthweight (BW) measurement recorded from health card for livebirths by place of delivery for India and its states, NFHS 5 by place of delivery. CI denotes confidence interval.**

|  | **Public health facility** | | | **Private health facility** | | | **Home delivery** | | |
| --- | --- | --- | --- | --- | --- | --- | --- | --- | --- |
|  | **Number of livebirths with BW available** | **Birthweight recorded from health card  N (%; 95% CI)** | **Birthweight recorded from mother’s recall  N (%; 95% CI)** | **Number of livebirths with BW available** | **Birthweight recorded from health card  N (%; 95% CI)** | **Birthweight recorded from mother’s recall  N (%; 95% CI)** | **Number of livebirths with BW available** | **Birthweight recorded from health card  N (%; 95% CI)** | **Birthweight recorded from mother’s recall  N (%; 95% CI)** |
| **India** | **1,44,655** | **89,373**  **(61.8; 61.5-62.0)** | **55,282 (38.2;37.9-38.5)** | **48,945** | **26,336 (53.8; 53.4-54.3)** | **22,609 (46.2;44.7-47.7)** | 15,666 | **8,656 (55.3;52.6-57.9)** | 7,010 (44.7;42.1-47.4) |
| **Less developed states** | **97,178** | **58,974**  **(60.7; 60.4-61.0)** | **38,204 (39.3;38.8-39.8)** | **22,280** | **11,012 (49.4; 48.8-50.1)** | **11,268 (50.6;47.9-53.2)** | 12,841 | **7,029 (54.7;51.2-58.2)** | 5,812 (45.3;41.8-48.8) |
| Arunachal Pradesh | 4,002 | 2,825 (70.6; 69.2-72.0) | 1,177 (29.4;26.8-32.1) | 232 | 153 (66.0; 59.8-72.1) | 79 (34.1;24.4-43.7) | 276 | 195 (70.7;62.2-79.1) | 81 (29.3;20.9-37.8) |
| Assam | 7,803 | 6,512 (83.5; 82.6-84.3) | 1,291 (16.5;14.8-18.3) | 986 | 797 (80.8; 78.4-83.3) | 189 (19.2;14.6-23.8) | 1,078 | 833 (77.3;72.6-82.0) | 245 (22.7;18.0-27.4) |
| Bihar | 11,438 | 5,460 (47.7; 46.8-48.7) | 5,978 (52.3;50.1-54.4) | 3,510 | 1,293 (36.8; 35.2-38.4) | 2,217 (63.2;59.8-66.5) | 1,288 | 599 (46.5;40.7-52.3) | 689 (53.5;47.7-59.3) |
| Chhattisgarh | 5,982 | 4,088 (68.3; 67.2-69.5) | 1,894 (31.7;28.7-34.7) | 1,043 | 701 (67.2; 64.4-70.1) | 342 (32.8;26.1-39.5) | 1,065 | 629 (59.1;52.1-66.0) | 436 (40.9;34.0-47.9) |
| Jharkhand | 5,712 | 3,833 (67.1; 65.9-68.3) | 1,879 (32.9;29.6-36.2) | 1,621 | 946 (58.4; 56.0-60.8) | 675 (41.6;32.8-50.5) | 1,266 | 887 (70.1;60.8-79.3) | 379 (29.9;20.7-39.2) |
| Madhya Pradesh | 12,796 | 7,077 (55.3; 54.5-56.2) | 5,719 (44.7;42.2-47.2) | 1,436 | 718 (50.0; 47.4-52.6) | 718 (50.0;39.2-60.8) | 926 | 458 (49.5;36.0-62.9) | 468 (50.5;37.1-64.0) |
| Manipur | 1,571 | 905 (57.6; 55.2-60.1) | 666 (42.4;34.9-49.9) | 561 | 294 (52.4; 48.3-56.5) | 267 (47.6;29.3-65.8) | 288 | 135 (46.9;21.4-72.3) | 153 (53.1;27.7-78.6) |
| Meghalaya | 3,044 | 2,043 (67.1; 65.5-68.8) | 1,001 (32.9;27.5-38.3) | 513 | 282 (55.0; 50.7-59.3) | 231 (45.0;25.5-64.5) | 1,919 | 795 (41.4;31.4-51.4) | 1,124 (58.6;48.6-68.6) |
| Mizoram | 1,743 | 958 (55.0; 52.6-57.3) | 785 (45.0;37.1-53.0) | 188 | 112 (59.6; 52.5-66.6) | 76 (40.4;7.9-73.0) | 281 | 173 (61.6;35.2-87.9) | 108 (38.4;12.1-64.8) |
| Nagaland | 943 | 600 (63.6; 60.6-66.7) | 343 (36.4;25.5-47.2) | 172 | 103 (59.9; 52.5-67.2) | 69 (40.1;4.6-75.6) | 403 | 216 (53.6;30.0-77.2) | 187 (46.4;22.8-70.0) |
| Odisha | 6,697 | 4,958 (74.0; 73.0-75.1) | 1,739 (26.0;22.1-29.8) | 1,013 | 677 (66.8; 63.9-69.7) | 336 (33.2;18.8-47.5) | 622 | 474 (76.2;59.6-92.8) | 148 (23.8;7.2-40.4) |
| Rajasthan | 11,150 | 6,226 (55.8; 54.9-56.8) | 4,924 (44.2;40.7-47.7) | 2,287 | 1,131 (49.5; 47.4-51.5) | 1,156 (50.5;40.0-61.1) | 342 | 166 (48.5;21.3-75.8) | 176 (51.5;24.2-78.7) |
| Sikkim | 515 | 364 (70.7; 66.7-74.6) | 151 (29.3;13.8-44.8) | 70 | 40 (57.1; 45.5-68.8) | 30 (42.9;0-103.7) | 22 | 14 (63.6;0-169.1) | 8 (36.4;0-141.8) |
| Tripura | 1,534 | 928 (60.5; 58.1-62.9) | 606 (39.5;29.6-49.4) | 212 | 112 (52.8; 46.1-59.6) | 100 (47.2;10.1-84.3) | 101 | 52 (51.5;0-105.3) | 49 (48.5;0-102.3) |
| Uttar Pradesh | 20,020 | 11,100 (55.5; 54.8-56.1) | 8,920 (44.6;41.7-47.4) | 7,683 | 3,296 (42.9; 41.8-44.0) | 4,387 (57.1;50.9-63.3) | 2,667 | 1,264 (47.4;36.8-58.0) | 1,403 (52.6;42.0-63.2) |
| Uttarakhand | 2,228 | 1,097 (49.2; 47.2-51.3) | 1,131 (50.8;41.8-59.7) | 753 | 357 (47.4; 43.8-51.0) | 396 (52.6;32.3-72.9) | 297 | 139 (46.8;14.4-79.2) | 158 (53.2;20.8-85.6) |
| **More developed states** | **45,287** | **29,137 (64.3; 63.9-64.8)** | **16,150 (35.7;33.7-37.6)** | **25,998** | **14,974 (57.6; 57.0-58.2)** | **11,022 (42.4;39.5-45.2)** | **2,782** | **1,603 (57.6;48.9-66.3)** | **1,179 (42.4;33.7-51.1)** |
| Andhra Pradesh | 1,405 | 650 (46.3; 43.7-48.9) | 755 (53.7;41.9-65.5) | 1,301 | 599 (46.0; 43.3-48.8) | 702 (54.0;50.6-57.3) | 74 | 24 (32.4;19.4-45.5) | 50 (67.6;54.5-80.6) |
| Delhi | 1,765 | 709 (40.2; 37.9-42.5) | 1,056 (59.8;49.2-70.4) | 837 | 318 (38.0; 34.7-41.3) | 519 (62.0;53.6-70.4) | 154 | 52 (33.8;14.7-52.8) | 102 (66.2;47.2-85.3) |
| Goa | 212 | 145 (68.4; 62.1-74.7) | 67 (31.6;1.9-61.3) | 154 | 105 (68.2; 60.8-75.6) | 49 (31.8;11.7-52.0) |  | - | - |
| Gujarat | 4,353 | 2,836 (65.1; 63.7-66.6) | 1,517 (34.8;28.0-41.7) | 4,622 | 2,712 (58.7; 57.3-60.1) | 1,,910 (41.3;37.2-45.5) | 554 | 334 (60.3;48.4-72.2) | 220 (39.7;27.8-51.6) |
| Haryana | 3,891 | 1,757 (45.2; 43.6-46.7) | 2,134 (54.8;47.1-62.6) | 2,412 | 1,057 (43.8; 41.8-45.8) | 1355 (56.2;50.1-62.3) | 202 | 86 (42.6;21.6-63.6) | 116 (57.4;36.4-78.4) |
| Himachal Pradesh | 1,833 | 1,295 (70.7; 68.6-72.7) | 538 (29.4;18.8-39.9) | 430 | 283 (65.8; 61.3-70.3) | 147 (34.2;19.7-48.7) | 235 | 136 (57.9;37.4-78.3) | 99 (42.1;21.7-62.6) |
| Jammu and Kashmir | 4,710 | 3,859 (81.9; 80.8-83.0) | 851 (18.1;12.4-23.7) | 275 | 219 (79.6; 74.9-84.4) | 56 (20.4;3.5-37.2) | 258 | 184 (71.3;51.8-90.8) | 74 (28.7;9.2-48.2) |
| Karnataka | 5,548 | 3,955 (71.3; 70.1-72.5) | 1,593 (28.7;22.5-35.0) | 2,438 | 1,629 (66.8; 65.0-68.7) | 809 (33.2;26.1-40.3) | 196 | 130 (66.3;41.1-91.5) | 66 (33.7;8.5-58.9) |
| Kerala | 950 | 676 (71.2; 68.3-74.0) | 274 (28.8;13.5-44.2) | 1,755 | 1,296 (73.9; 71.8-75.9) | 459 (26.2;18.1-34.2) | 5 | 3 (60.0;0-229.1) | 2 (40.0;0-209.1) |
| Maharashtra | 5,676 | 3,523 (62.1; 60.8-63.3) | 2,153 (37.9;31.1-44.8) | 3,091 | 1,671 (54.1; 52.3-55.8) | 1,420 (45.9;38.4-53.5) | 373 | 190 (50.9;29.1-72.8) | 183 (49.1;27.2-70.9) |
| Punjab | 3,006 | 1,480 (49.2; 47.5-51.0) | 1,526 (50.8;40.9-60.6) | 2,135 | 1,067 (50.0; 47.9-52.1) | 1,068 (50.0;39.3-60.7) | 178 | 82 (46.1;9.1-83.0) | 96 (53.9;17.0-90.9) |
| Tamil Nadu | 4,329 | 2,938 (67.9; 66.5-69.1) | 1,391 (32.1;24.3-39.9) | 2,103 | 1,401 (66.6; 64.6-68.6) | 702 (33.4;22.6-44.1) | 22 | 17 (77.3;0-170.8) | 5 (22.7;0-116.2) |
| Telangana | 3,596 | 1,971 (54.8; 53.2-56.4) | 1,625 (45.2;35.9-54.5) | 3,412 | 1,780 (52.2; 50.5-53.9) | 1,632 (47.8;38.7-56.9) | 169 | 85 (50.3;9.4-91.2) | 84 (49.7;8.8-90.6) |
| West Bengal | 4,013 | 3,343 (83.3; 82.2-84.5) | 670 (16.7;10.0-23.4) | 1,033 | 839 (81.2; 78.8-83.6) | 194 (18.8;5.0-32.6) | 362 | 280 (77.3;52.4-102.3) | 82 (22.7;0-47.6) |
