# Supplementary material for: Increase in birthweight coverage of neonatal deaths is needed to monitor low birthweight prevalence in India: lessons from the National Family Health Survey
Source: BMC Pregnancy Childbirth. 2023 Jul 29;23:545. doi: 10.1186/s12884-023-05865-2 (PMC10386228; doi:10.1186/s12884-023-05865-2)
Supplement: Supplementary file 3 — Additional file 3. Distribution of the data source for birthweight by the year of birth for livebirths, India. [file 12884_2023_5865_MOESM3_ESM.docx]

1. **Distribution of the data source for birthweight by the year of birth for livebirths, India.**
