# Supplementary material for: Increase in birthweight coverage of neonatal deaths is needed to monitor low birthweight prevalence in India: lessons from the National Family Health Survey
Source: BMC Pregnancy Childbirth. 2023 Jul 29;23:545. doi: 10.1186/s12884-023-05865-2 (PMC10386228; doi:10.1186/s12884-023-05865-2)
Supplement: Supplementary file 4 — Additional file 4. Percent of heaping (birthweight documented at 2500 or 3000 or 3500gms) in birthweight, India and its states, NFHS 5. CI denotes confidence interval. [file 12884_2023_5865_MOESM4_ESM.docx]

1. **Percent of heaping (birthweight documented at 2500 or 3000 or 3500gms) in birthweight, India and its states, NFHS 5. CI denotes confidence interval.**

|  | **Percent of birthweight recorded as** | | | |
| --- | --- | --- | --- | --- |
|  | **2500g  N (%; 95% CI)** | **3000g  N (%; 95% CI)** | **3500g  N (%; 95% CI)** | **2500, 3000, or 3500g  N (%; 95% CI)** |
| **India** | **41,044 (19.6; 19.4-19.8)** | **49,663 (23.7; 23.6-23.9)** | **18,024 (8.6; 8.5-8.7)** | **1,08,731 (52.0; 51.7-52.2)** |
| **Less developed states** | **26,277 (19.9; 19.7-20.1)** | **31,679 (24.0; 23.7-24.2)** | **11,331 (8.6; 8.4-8.7)** | **69,287 (52.4; 52.1-52.6)** |
| Arunachal Pradesh | 328 (7.3; 6.5-8.0) | 1,027 (22.8; 21.6-24.0) | 441 (9.8; 8.9-10.7) | 1,796 (39.8; 38.4-41.3) |
| Assam | 1,640 (16.6; 15.9-17.4) | 1,928 (19.5; 18.8-20.3) | 501 (5.1; 4.6-5.5) | 4,069 (41.2; 40.3-42.2) |
| Bihar | 3,332 (20.5; 19.9-21.1) | 4,172 (25.7; 25.0-26.4) | 1,757 (10.8; 10.3-11.3) | 9,261 (57.0; 56.3-57.8) |
| Chhattisgarh | 1,889 (23.4; 22.4-24.3) | 2,531 (31.3; 30.3-32.3) | 676 (8.4; 7.8-9.0) | 5,096 (63.0; 61.9-64.0) |
| Jharkhand | 1,813 (21.1; 20.2-22.0) | 2,260 (26.3; 25.4-27.2) | 725 (8.4; 7.8-9.0) | 4,798 (55.8; 54.8-56.9) |
| Madhya Pradesh | 3,552 (23.4; 22.8-24.1) | 3,775 (24.9; 24.2-25.6) | 1,263 (8.3; 7.9-8.8) | 8,590 (56.7; 55.9-57.5) |
| Manipur | 153 (6.3; 5.4-7.3) | 570 (23.6; 21.9-25.3) | 303 (12.5; 11.2-13.8) | 1,026 (42.4; 40.4-44.4) |
| Meghalaya | 359 (6.6; 5.9-7.2) | 978 (17.9; 16.9-18.9) | 395 (7.2; 6.5-7.9) | 1,732 (31.6; 30.4-32.9) |
| Mizoram | 66 (3.0; 2.3-3.7) | 356 (16.1; 14.6-17.6) | 261 (11.8; 10.5-13.1) | 683 (30.9; 29.0-32.8) |
| Nagaland | 92 (6.1; 4.9-7.3) | 543 (35.8; 33.4-38.2) | 227 (15.0; 13.2-16.8) | 862 (56.8; 54.3-59.3) |
| Odisha | 1,236 (14.8; 14.1-15.6) | 1,029 (12.4; 11.6-13.1) | 340 (4.1; 3.7-4.5) | 2,605 (31.3; 30.3-32.3) |
| Rajasthan | 3,122 (22.7; 22.0-23.4) | 3,359 (24.4; 23.7-25.1) | 944 (6.9; 6.4-7.3) | 7,425 (53.9; 53.1-54.7) |
| Sikkim | 41 (6.8; 4.8-8.8) | 105 (17.3; 14.3-20.3) | 60 (9.9; 7.5-12.3) | 206 (33.9; 30.2-37.7) |
| Tripura | 273 (14.8; 13.2-16.4) | 309 (16.7; 15.0-18.4) | 118 (6.4; 5.3-7.5) | 700 (37.9; 35.7-40.1) |
| Uttar Pradesh | 7,477 (24.6; 24.1-25.1) | 7,826 (25.8; 25.3-26.3) | 3,008 (10.0; 9.6-10.2) | 18,311 (60.3; 59.7-60.8) |
| Uttarakhand | 904 (27.6; 26.1-29.1) | 911 (27.8; 26.3-29.3) | 312 (9.5; 8.5-10.5) | 2,127 (64.9; 63.3-66.5) |
| **More developed states** | **14,265(19.3; 19.0-19.5)** | **17,414 (23.5; 23.2-23.8)** | **6,453 (8.7; 8.5-8.9)** | **38<132 (51.5; 51.1-51.8)** |
| Andhra Pradesh | 603 (21.7; 20.2-23.2) | 928 (33.4; 31.6-35.1) | 329 (11.8; 10.6-13.0) | 1,860 (66.9; 65.2-68.7) |
| Delhi | 467 (17.0; 15.5-18.4) | 456 (16.6; 15.2-17.9) | 262 (9.5; 8.4-10.6) | 1,185 (43.0; 41.2-44.9) |
| Goa | 65 (17.8; 13.8-21.7) | 53 (14.5; 10.9-18.1) | 23 (6.3; 3.8-8.8) | 141 (38.5; 33.5-43.5) |
| Gujarat | 1,978 (20.8; 19.9-21.6) | 1,698 (17.8; 17.1-18.6) | 828 (8.7; 8.1-9.3) | 4,504 (47.3; 46.3-48.3) |
| Haryana | 1,458 (22.4; 22.0-23.4) | 1,378 (21.2; 20.2-22.2) | 629 (9.7; 9.0-10.4) | 3,465 (53.3; 52.1-54.5) |
| Himachal Pradesh | 454 (18.2; 16.7-19.7) | 508 (20.3; 18.8-21.9) | 212 (8.5; 7.4-9.6) | 1,174 (47.0; 45.0-49.0) |
| Jammu and Kashmir | 993 (18.9; 17.9-20.0) | 1,807 (34.5; 33.2-35.8) | 435 (8.3; 7.6-9.04) | 3,235 (61.7; 60.4-63.0) |
| Karnataka | 1,709 (20.9; 20.0-21.8) | 2,107 (25.8; 24.8-26.7) | 898 (11.0; 10.3-11.7) | 4,714 (57.6; 56.5-58.7) |
| Kerala | 174 (6.4; 5.5-7.3) | 272 (10.0; 8.9-11.2) | 121 (4.5; 3.7-5.2) | 567 (20.9; 19.4-22.5) |
| Maharashtra | 1,978 (21.6; 20.8-22.5) | 2,591 (28.4; 27.4-29.3) | 808 (8.8; 8.3-9.4) | 5,377 (58.8; 57.8-59.8) |
| Punjab | 1,335 (25.1; 23.9-26.3) | 1,419 (26.7; 25.5-27.9) | 525 (9.9; 9.1-10.7) | 3,279 (61.7; 60.3-63.0) |
| Tamil Nadu | 748 (11.6; 10.8-12.4) | 840 (13.0; 12.2-13.8) | 364 (5.6; 5.1-6.2) | 1,952 (30.3; 29.1-31.4) |
| Telangana | 1,532 (21.4; 20.4-22.3) | 2,535 (35.3; 34.2-36.4) | 758 (10.6; 9.9-11.3) | 4,825 (67.2; 66.1-68.3) |
| West Benga | 771 (14.3; 13.3-15.2) | 822 (15.2; 14.2-16.2) | 261 (4.8; 4.3-5.4) | 1,854 (34.3; 33.0-35.6) |
