# Supplementary material for: Increase in birthweight coverage of neonatal deaths is needed to monitor low birthweight prevalence in India: lessons from the National Family Health Survey
Source: BMC Pregnancy Childbirth. 2023 Jul 29;23:545. doi: 10.1186/s12884-023-05865-2 (PMC10386228; doi:10.1186/s12884-023-05865-2)
Supplement: Supplementary file 5 — Additional file 5. Percent of heaping (birthweight documented at 2500 or 3000 or 3500gms) by documentation source, India and its states, NFHS-5. CI denotes confidence interval. [file 12884_2023_5865_MOESM5_ESM.docx]

1. **Percent of heaping (birthweight documented at 2500 or 3000 or 3500gms) by documentation source, India and its states, NFHS-5.**

**CI denotes confidence interval.**

|  | **Birthweight available from health card (N)** | **% of heaping in health card N (%; 95% CI)** | **Heaping** | **Birthweight available from mother’s recall (N)** | **% of heaping in mother’s recall N (%; 95% CI)** | **Heaping** |
| --- | --- | --- | --- | --- | --- | --- |
| **India** | **1,24,365** | **62,161 (50.0; 49.7-50.3)** | No | **84,901** | **46,570 (54.9; 54.5-55.2)** | No |
| **Less developed states** | **77,015** | **38,788 (50.4; 50.0-50.7)** | No | **55,284** | **30,499 (55.2; 54.8-55.6)** | No |
| Arunachal Pradesh | 3,173 | 1,245 (39.2; 37.5-40.9) | No | 1,337 | 551 (41.2; 38.6-43.9) | No |
| Assam | 8,142 | 3,429 (42.1; 41.0-43.2) | No | 1,725 | 640 (37.1; 34.8-39.4) | No |
| Bihar | 7,352 | 3,957 (53.8; 52.7-55.0) | No | 8,884 | 5,304 (59.7; 58.7-60.7) | Yes |
| Chhattisgarh | 5,418 | 3,363 (62.1; 60.8-63.4) | Yes | 2,672 | 1,733 (64.9; 63.0-66.7) | Yes |
| Jharkhand | 5,666 | 3,074 (54.3; 53.0-55.6) | No | 2,933 | 1,724 (58.8; 57.0-60.6) | Yes |
| Madhya Pradesh | 8,253 | 4,540 (55.0; 53.9-56.1) | No | 6905 | 4,050 (58.7; 57.5-59.8) | Yes |
| Manipur | 1334 | 557 (41.8; 39.1-44.4) | No | 1086 | 469 (43.2; 40.2-46.1) | No |
| Meghalaya | 3,120 | 947 (30.4; 28.7-32.0) | No | 2,356 | 785 (33.3; 31.4-35.2) | No |
| Mizoram | 1,243 | 387 (31.1; 28.6-33.7) | No | 969 | 296 (30.6; 27.6-33.4) | No |
| Nagaland | 919 | 513 (55.8; 52.6-59.0) | Yes | 599 | 349 (58.3; 54.3-62.2) | Yes |
| Odisha | 6,109 | 1,907 (31.2; 30.1-32.4) | No | 2,223 | 698 (31.4; 29.5-33.3) | No |
| Rajasthan | 7,523 | 3,970 (52.8; 51.6-53.9) | No | 6,256 | 3,455 (55.2; 54.0-56.5) | No |
| Sikkim | 418 | 134 (32.1; 27.6-36.5) | No | 189 | 72 (38.1; 31.2-45.0) | No |
| Tripura | 1,092 | 401 (36.7; 33.9-39.6) | No | 755 | 299 (39.6; 36.1-43.1) | No |
| Uttar Pradesh | 15,660 | 9,333 (59.6; 58.8-60.4) | Yes | 14,710 | 8,978 (61.0; 60.2-61.8) | Yes |
| Uttarakhand | 1,593 | 1,031 (64.7; 62.4-67.1) | No | 1,685 | 1,096 (65.0; 62.8-67.3) | Yes |
| **More developed states** | **45,716** | **22,667 (49.6; 49.1-50.0)** | No | **28,351** | **15,465 (54.6; 54.0-55.1)** | No |
| Andhra Pradesh | 1,273 | 883 (69.4; 66.8-71.9) | Yes | 1,507 | 977 (64.8; 62.4-67.2) | Yes |
| Delhi | 1079 | 411 (38.1; 35.2-41.0) | No | 1,677 | 774 (46.2; 43.8-48.5) | No |
| Goa | 250 | 87 (34.8; 28.9-40.7) | No | 116 | 54 (46.6; 37.4-55.7) | No |
| Gujarat | 5,882 | 2,526 (42.9; 41.7-44.2) | No | 3,647 | 1,978 (54.2; 52.6-55.9) | No |
| Haryana | 2,900 | 1,534 (52.9; 51.1-54.7) | No | 3,605 | 1,931 (53.6; 51.9-55.2) | No |
| Himachal Pradesh | 1,714 | 766 (44.7; 42.3-47.0) | No | 784 | 408 (52.0; 48.5-55.5) | No |
| Jammu and Kashmir | 4,262 | 2,633 (61.8; 60.3-63.2) | Yes | 981 | 602 (61.4; 58.3-64.4) | Yes |
| Karnataka | 5,714 | 3,295 (57.7; 56.4-58.9) | Yes | 2,468 | 1,419 (57.5; 55.5-59.4) | Yes |
| Kerala | 1,975 | 382 (19.3; 17.6-21.1) | No | 735 | 185 (25.2; 22.0-28.3) | No |
| Maharashtra | 5,384 | 3,212 (59.7; 58.3-61.0) | Yes | 3,756 | 2,165 (57.6; 56.1-59.2) | Yes |
| Punjab | 2,629 | 1,592 (60.6; 58.7-62.4) | Yes | 2,690 | 1,687 (62.7; 60.9-64.5) | Yes |
| Tamil Nadu | 4,356 | 1,263 (29.0; 27.6-30.3) | No | 2,098 | 689 (32.8; 30.8-34.9) | No |
| Telangana | 3,836 | 2,575 (67.1; 65.6-68.6) | Yes | 3,341 | 2,250 (67.4; 65.8-68.9) | Yes |
| West Bengal | 4,462 | 1,508 (33.8; 32.4-35.2) | No | 946 | 1. (36.6; 33.5-39.6) | No |
