# Supplementary material for: Increase in birthweight coverage of neonatal deaths is needed to monitor low birthweight prevalence in India: lessons from the National Family Health Survey
Source: BMC Pregnancy Childbirth. 2023 Jul 29;23:545. doi: 10.1186/s12884-023-05865-2 (PMC10386228; doi:10.1186/s12884-023-05865-2)
Supplement: Supplementary file 6 — Additional file 6. Correlation of heaping in birthweight (BW) by coverage of birthweight measurement from data source for India, NFHS 5 (2019-21). [file 12884_2023_5865_MOESM6_ESM.docx]

# **Correlation of heaping in birthweight (BW) by coverage of birthweight measurement from data source for India, NFHS 5 (2019-21)**
